# The role of inflammation in HPV infection of the Oesophagus

**DOI:** 10.1186/1471-2407-13-185

**Published:** 2013-04-09

**Authors:** Georgia Schäfer, Siti Kabanda, Beverly van Rooyen, Martina Bergant Marušič, Lawrence Banks, M Iqbal Parker

**Affiliations:** 1 International Centre for Genetic Engineering and Biotechnology, Cape Town, South Africa; 2 Division of Medical Biochemistry, University of Cape Town, South Africa and the MRC/UCT Oesophageal Cancer Research Unit, Cape Town, South Africa; 3 International Centre for Genetic Engineering and Biotechnology (ICGEB), Trieste, Italy

**Keywords:** HPV, Cytokines, Receptors, Oesophageal cancer

## Abstract

**Background:**

Several human cancers are known to be associated with inflammation and/or viral infections. However, the influence of tumour-related inflammation on viral uptake is largely unknown. In this study we used oesophageal squamous cell carcinoma (OSCC) as a model system since this type of cancer is associated with chronic irritation, inflammation and viral infections. Although still debated, the most important viral infection seems to be with Human Papillomavirus (HPV). The present study focused on a possible correlation between inflammation, OSCC development and the influence of HPV infection.

**Methods:**

A total of 114 OSCC biopsies and corresponding normal tissue were collected at Groote Schuur Hospital and Tygerberg Hospital, Cape Town (South Africa), that were subjected to RNA and DNA isolation. RNA samples were analysed by quantitative Light Cycler RT-PCR for the expression of selected genes involved in inflammation and infection, while conventional PCR was performed on the DNA samples to assess the presence of integrated viral DNA. Further, an in vitro infection assay using HPV pseudovirions was established to study the influence of inflammation on viral infectivity using selected cell lines.

**Results:**

HPV DNA was found in about 9% of OSCC patients, comprising predominantly the oncogenic type HPV18. The inflammatory markers IL6 and IL8 as well as the potential HPV receptor ITGA6 were significantly elevated while IL12A was downregulated in the tumour tissues. However, none of these genes were expressed in a virus-dependent manner. When inflammation was mimicked with various inflammatory stimulants such as benzo-α-pyrene, lipopolysaccharide and peptidoglycan in oesophageal epithelial cell lines in vitro, HPV18 pseudovirion uptake was enhanced only in the benzo-α-pyrene treated cells. Interestingly, HPV pseudovirion infectivity was independent of the presence of the ITGA6 receptor on the surface of the tested cells.

**Conclusion:**

This study showed that although the carcinogen benzo-α-pyrene facilitated HPV pseudovirion uptake into cells in culture, HPV infectivity was independent of inflammation and seems to play only a minor role in oesophageal cancer.

## Background

Viral infections are known to contribute to the development of several human cancers, the best known being the association of Human Papillomavirus (HPV) with cervical cancer [1]. Tumorigenesis can be induced by infectious agents through the induction of chronic inflammation, cellular transformation by oncogene insertion, inhibition of tumour suppressors and induction of immunosuppression [2]. While the consequences of inflammation on tumour initiation and progression are well studied [3,4], the relationship between inflammation and viral infections in carcinogenesis is much less understood.

The incidence of oesophageal cancer is highly variable depending on geographical and ethnic parameters. High risk areas include the so-called Asian oesophageal cancer belt from eastern Turkey, through Iraq, Iran and into Western and Northern China as well as Eastern-Southern Africa, South America and southern areas of France [5]. Oesophageal cancer is the most common cancer among black South African men, and is second to cervical carcinoma in black women [6]. There are two major types of oesophageal cancer: squamous cell carcinoma (OSCC) and adenocarcinoma, the former being the predominant form in developing countries. While chronic, frequent reflux of gastric acid into the distal oesophagus is considered as the primary factor underlying most cases of oesophageal adenocarcinoma affecting predominantly white populations [7], oesophageal squamous-cell carcinomas are more frequent among Black and Asian populations and are most strongly associated with smoking, alcohol intake, lack of fresh fruit and vegetables in the diet, and infections [8,9]. Although still debated due to conflicting results, infection with HPV that was shown to be correlated to nasopharyngeal cancer [10] is thought to be an aetiological factor in OSCC depending on the geographical region with prevalence ranging from 0% to 71% [10-16]. This is in contrast to other types of cancer that are clearly related to HPV infection such as cervical cancers [1]. It is assumed that only oesophageal cancers originating from high-incidence geographic areas are associated with HPV infection, particularly with the high-risk types 16 and 18 [11,17-20].

Previous studies from our laboratory revealed a correlation between HPV infection and oesophageal cancer in patients from the Transkei (Eastern Cape), a particularly high-risk area in South Africa: 46% were infected with HPV with the predominant type being HPV11 [14,21]. In the present study we investigated the role of HPV infection of OSCC patients from the Western Cape (South Africa) and specifically focussed on a possible correlation between inflammation and OSCC, the influence of HPV infection for tumour-associated inflammation, and the role of inflammation for virus uptake. To our knowledge, there is little information on the potential role of chronic inflammation in the aetiology of HPV-associated OSCC, with a possible connection being described for a subset of head and neck squamous cell carcinoma (HNSCC) [22].

The genes that were selected in the present study for analysis of altered gene expression in the tumour samples were p16 as an HPV type-independent cellular correlate for increased HPV oncogene expression [23], potential receptors for HPV (ITGA6, SDC) [24] and representative genes of cancer-associated inflammation (IL6, IL8, IL12A, IFNGR1, TNFA1P1) [25]. Additionally, the effects of benzo-α-pyrene (BαP), one of the most important tobacco smoke carcinogens [26-29], as well as of peptidoglycan (PEP) and lipopolysaccharide (LPS), both known to induce IL6 and IL8 [30-33], were tested.

We found that the presence of HPV DNA in OSCC tissues was rather low and appeared to be independent of inflammation or the presence of the potential HPV receptor ITGA6 [34]. Virus uptake was only increased in vitro when the cells were treated with benzo-α-pyrene, one of the most abundant carcinogens in tobacco smoke.

## Methods

### Tissue collection

A total of 114 histopathologically-confirmed OSCC biopsies together with corresponding adjacent normal tissue samples were collected at both Groote Schuur Hospital and Tygerberg Hospital, Cape Town, South Africa, between 2008 and 2011. Patients were from different ethnic groups with 66% being black (Xhosa-speaking), 32% of mixed ancestry, and 2% whites. Informed consent was obtained from all participants, while ethical approval for this study was obtained from the Ethics Committee of the University of Cape Town. Samples were stored in RNAlater solution (Qiagen) at −80°C until used for nucleic acid extraction.

### Cell culture

The immortalised human oesophageal epithelial cell line EPC2-hTERT [35] was grown in Keratinocyte SFM medium (Invitrogen) supplemented with 50 μg/ml bovine pituitary extract (Invitrogen), 1 ng/ml human EGF (Invitrogen), 100 U/ml penicillin and 100 μg/ml streptomycin. WHCO1 (human OSCC cell line) [36], HaCaT (spontaneously immortalized human keratinocyte cell line and natural host for HPV [37], ATCC), C33A (human HPV negative cervical cancer cell line, ATCC) and 293TT cells (virus packaging cell line established from primary embryonal human kidney cells transformed with modified human adenovirus) [38,39] were grown in DMEM (Gibco Life Technologies) supplemented with 10% heat-inactivated fetal calf serum (Gibco Life Technologies), 100U/ml penicillin and 100 μg/ml streptomycin. CHO-K1 (Chinese hamster epithelial-like cells, ATCC) and pgsD-677 cells (heparin sulfate deficient cells derived from CHO-K1, ATCC) were cultured in Ham`s F-12 K medium (Gibco Life Technologies) supplemented with 10% heat-inactivated fetal calf serum, 100U/ml penicillin and 100 μg/ml streptomycin.

### Nucleic acid extraction and PCR

Frozen biopsies were cut in half for parallel RNA and genomic DNA extraction with the TRIzol reagent (Invitrogen) and the QIAamp Mini Kit (Qiagen), respectively. For PCR amplification of the HPV L1 gene, 100 ng DNA was used in a nested PCR reaction containing the degenerate consensus primers MY09/MY11 followed by PCR with the primer set GP5+/GP6+ as described previously [14] using the FastStart Taq DNA polymerase (Roche). The presence of EBV DNA was detected similarly by targeting the virus capsid antigen p23 region by nested PCR using the primer set p23-1/p23-2 followed by PCR with the primer set p23-3/p23-4 [40]. Amplification of human β-actin was used as an internal control (see Table 1 for all primer sequences and PCR product sizes). The integrity of the PCR products was visualised by agarose gel electrophoresis. The PCR products from all HPV positive samples were subjected to DNA sequence analysis to determine the HPV type. For quantitative RT-PCR, RNA extracted from the biopsies was reverse transcribed using the ImProm-II™ Reverse Transcription System (Promega). cDNA generated from 1 μg of total RNA was used for quantitative PCR with the KAPA SYBR®FAST qPCR Kit (Kapa Biosystems) on a LightCycler®480II System (Roche). Products were amplified with the primers listed in Table 1 under the following conditions: 1 min 95°C, 1 min 55°C, 30s 72°C. Results were analysed using the 2^-ΔΔCт^ method [41], normalised to GAPDH expression and represented as x-fold increase in an individual tumour sample (T) compared to the corresponding non-cancerous normal tissue (N) from the same patient. Statistics were done to compare the significance of gene expression in T versus N using the 2-tailed non-parametric Mann–Whitney test. All primers were tested for amplification of the correct products by conventional PCR using appropriate tissue DNA.

**Table 1:** Primers used in this study

**Primer name**	**Sequence (5**^**′**^**-3**^**′**^**)**	**Length (bp)**
pGL3 forw	CGGGCGCGGTCGGTAAAGT	380
pGL3 rev	AACAACGGCGGCGGGAAGT	
p16 forw	GCCACTCTCACCCGACCCGT	130
p16 rev	TCAGCCAGGTCCACGGGCAGA	
ITGA6 forw	GCCAGCAAGGTGTAGCAGCTA	101
ITGA6 rev	TTGCTCTACACGAACAATCCCTTT	
SDC1 forw	CCCCGTTTCTGGTGGTCT	175
SDC1 rev	TGTCTGAAGGCTGAGTCCC	
CD21 forw	GCCGACACGACTACCAACC	150
CD21 rev	AGCAAGTAACCAGATTCACAGC	
IL8 forw	GAGAGTGATTGAGAGTGGACCAC	111
IL8 rev	CACAACCCTCTGCACCCAGTTT	
IL6	SABiosciences (PPH00560B)	160
IL12A	SABiosciences (PPH00544B)	82
IFNGR1	SABiosciences (PPH00871B)	112
TNFA1P1	SABiosciences (PPH01176A)	101
GAPDH forw	GGCTCTCCAGAACATCATCC	192
GAPDH rev	GCCTGCTTCACCACCTTC	
MY09^a, b^	CGTCCMARRGGAWACTGATC	452
MY11	GCMCAGGGWCATAAYAATGG	
GP5+	TTTGTTACTGTGGTAGATACTAC	150
GP6+	GAAAAATAAACTGTAAATCATATTC	
EBV-P23-1	CAGCTCCACGCAAAGTCAGATTG	482
EBV-P23-2	ATCAGAAATTTGCACTTTCTTTGC	
EBV-P23-3	TTGACATGAGCATGGAAGAC	363
EBV-P23-4	CTCGTGGTCGTGTTCCCTCAC	
β-actin forw	ATCATGTTTGAGACCTTCAA	320
β-actin rev	CATCTCTTGCTCGAAGTCCA	

^a^ M, A+C: R, A+G; W, A+T; Y, C+T;

^b^ degenerate primers targeting the HPV late structural protein L1.

### HPV pseudovirion production, quantification and labelling

HPV16 and HPV18 pseudovirions encapsidating the luciferase reporter gene plasmid pGL3-control (Promega) were generated in 293TT cells as previously described [38,42]. The plasmid pXULL, coexpressing both codon-optimized HPV16 L1 and L2, was generously provided by Michelle Ozbun (University of New Mexico), while the plasmid HPV18-L1/L2 was kindly provided by Samuel K. Campos (University of Arizona). Purity and L1/L2 protein content were determined by SDS-PAGE and subsequent staining with the Pierce® Silver Stain Kit (Thermo Scientific). For PsVs labelling with a fluorochrome that is only activated after cell entry [43], 20 μg of purified PsVs were mixed with 200 μM Oregon Green®488 carboxylic acid diacetate succinimidyl ester (Molecular Probes) in a final volume of 500 μl HSB (pH 7.5) and incubated for 24 h at room temperature in the dark rotating. Unincorporated fluorochrome was removed by washing and concentrating the labelled PsVs using Amicon Ultra-4 (100,000 kDa MWCO) filter devices (Millipore). To assess the amount of encapsidated pGL3 plasmid DNA (viral genome equivalent, vge), all purified pseudovirion preparations were subjected to quantitative Light Cycler PCR using the primers pGL3 forw/pGL3 rev (Table 1).

### HPV pseudovirion infection assays

Cells were seeded in 12-well plates at a density of 5×10^4^ per well, and the following day cells were infected with a vge of approximately 100 pseudovirion particles per cell. For neutralisation assays, PsVs were incubated with the neutralising antibodies H16.V5 and H18.J4, respectively (generously provided by Neil D. Christensen, Pennsylvania State University College of Medicine) for 1 h at 4°C before adding to the cells. Where indicated, cells were stimulated with either 200 ng/ml IFNγ (R&D systems), 100 ng/ml IL6 (R&D systems), 100 ng/ml IL8 (R&D systems), 50 ng/ml IL12 (R&D systems), 5 μg/ml LPS (lipopolysaccharide) from *E*. *coli* 0127: B8 (Sigma), 50 μg/ml PEP (peptidoglycan) from *S*.*aureus* (Sigma) or 10 μM BαP (benzo-α-pyrene) (Sigma) for 24 h before addition of the PsVs. 48 h after infection, cells were harvested and luciferase activity was measured using the Luciferase Assay System kit (Promega) with the Fluoroscan Ascent FL (Thermo Fisher Scientific) according to the manufacturer’s instructions. Luciferase data were normalized to cell numbers by fluorescently staining an independent set of samples with 5 μM CellTrace™ Oregon Green® 488 (Molecular Probes) in PBS for 5 min at room temperature. All experiments were performed in duplicates, repeated at least three times and calculated as means ± S.D. with the Student’s *t* test used for determination of statistical significances.

To assess the uptake of PsVs that were labelled with Oregon Green®488 carboxylic acid diacetate succinimidyl ester (Molecular Probes) as described above, cells were seeded on coverslips in 6-well plates at a density of 2×10^5^ per well, grown overnight and infected with a vge of approximately 500 pseudovirion particles per cell for 2 h. Cells were washed, fixed with 4% paraformaldehyde and mounted in mowial®4-88 reagent (Calbiochem) on glass slides. Fluorescent cells were visualised using an Olympus IX81S8F fluorescent microscope at 40x magnification.

### Flow cytometry

Cells were prepared for FACS analysis as previously described [44]. Rat anti-human ITGA6 (clone NKI-GoH3, AbD Serotec) antibody together with R-Phycoerythrin-conjugated donkey anti-rat IgG were used to detect ITGA6 cell surface expression using a FACSCalibur (Becton Dickinson) together with the software CellQuest.

## Results

### Viral infection and cellular gene expression in OSCC tissue

**Figure 1:**
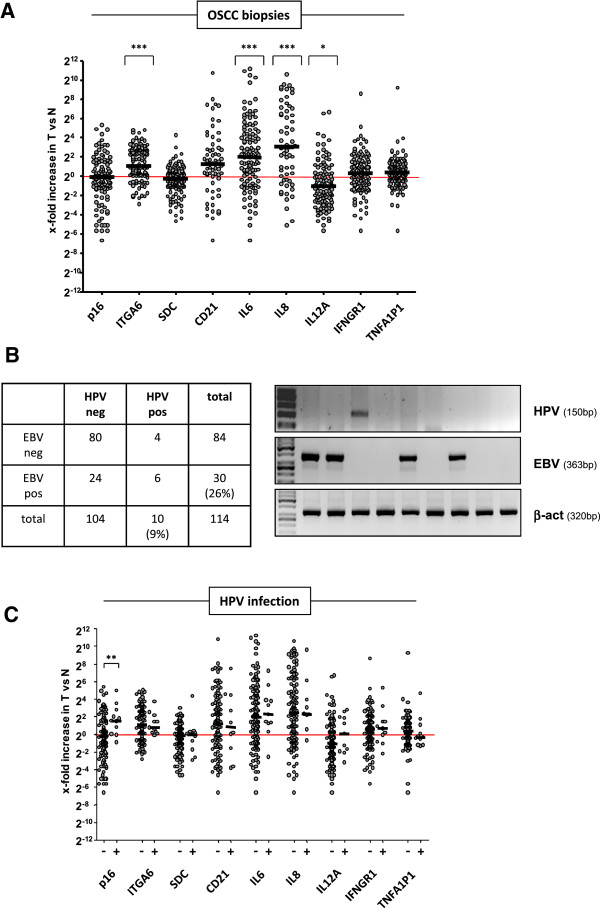
**Gene expression profile and infection with HPV and EBV in OSCC tissue.** (**A**) A total of 114 OSCC biopsies (T) was tested for the expression of the indicated genes by quantitative Light Cycler RT-PCR which is represented as x-fold increase compared to non-cancerous normal (N) tissue from the same patient. GAPDH expression served as internal control and was used for normalisation of gene expression data. The baseline at 2^0^ indicates no difference in expression between N and T. Statistics were done to compare the significance of gene expression in T versus N using the 2-tailed non-parametric Mann–Whitney test. Shown are the median values to account for outliers. *, p<0.05; **, p<0.01; ***, p<0.001. (**B**) Representative agarose gel showing conventional PCR results for the detection of HPV and EBV sequences in the OSCC genomic DNA. Separate amplification of ß-actin served as control for DNA integrity. The table summarizes the total number of OSCC samples that were positive or negative for HPV and EBV, respectively. Percentages in brackets illustrate HPV and EBV infection, respectively. (**C**) Quantitative Light Cycler RT-PCR data from (**A**) are depicted depending on HPV infection. Note that only upregulation of p16 in HPV positive OSCC samples reached statistical significance compared to HPV negative samples.

Based on the previous observation that 46% of OSCC patients from the Eastern Cape (South Africa), a particularly high-incidence geographic area for OSCC, contained integrated HPV DNA, predominantly HPV11 [14], we conducted a similar study in the Western Cape of South Africa. A total of 114 biopsies collected from histopathologically-confirmed OSCC patients were used for the analysis of the expression profile of selected genes and the presence of integrated HPV. Parallel detection of Epstein-Barr Virus (EBV) DNA served as technical control for the applied PCR-based detection of viral DNA as EBV is known to be associated with nasopharyngeal and gastric cancer [16,45] and possibly oesophageal cancer [10,13]. We found that the potential HPV receptor ITGA6 [24] and the EBV receptor CD21 EBV [46], that was included as control for a viral receptor, were up-regulated in the tumour samples compared to corresponding normal tissue, though CD21 did not reach statistical significance (Figure 1A). Moreover, the pro-inflammatory mediators IL6 and IL8 were significantly overexpressed in the tumours, while IL12A was significantly downregulated (Figure 1A). When OSCC genomic DNA was analysed for viral gene integration by conventional nested PCR, 9% of patients were found to be positive for HPV while 26% were EBV positive (Figure 1B) with the corresponding normal tissue being negative for HPV and EBV, respectively. The products derived from the HPV-PCR were subjected to DNA sequence analysis which revealed that the majority (8/10) of the HPV positive OSCC biopsies contained the high-risk type HPV18 DNA. Statistical analysis of the data shown in Figure 1A revealed that there was no significant difference in the expression of the cellular genes between tissues that tested positive or negative for HPV DNA (Figure 1C), except for significantly upregulated p16 in HPV positive OSCC tissues as would be expected [23] (Figure 1C).

### Infection of oesophageal epithelial and cancer cell lines with HPV-PsVs

**Figure 2:**
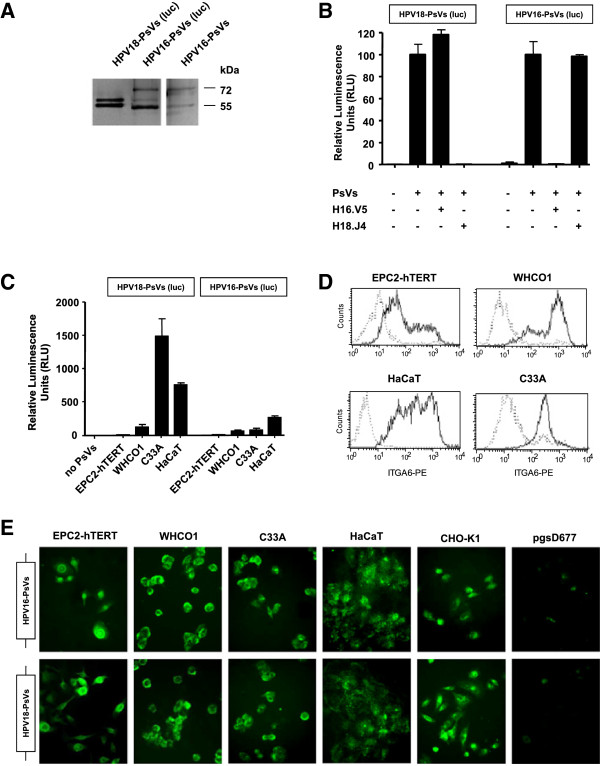
**Infection with HPV-PsVs in vitro.** Luciferase reporter gene encapsidating HPV-PsVs composed of HPV18-L1/L2 or HPV16-L1/L2 envelope proteins, respectively, were prepared as described previously [38,42]. (**A**) Purity of the preparations was analyzed by SDS-PAGE followed by silver staining. Note that the additional band at approximately 60 kDa in the HPV16-PsVs (luc) preparation is the silver-stained pGL3 plasmid which is not present in the HPV16-PsVs preparation that does not encapsidate any DNA. (**B**) In order to test functionality of the preparations, HaCaT cells were infected with HPV18-PsVs (luc) or HPV16-PsVs (luc) using a vge of 100 pseudovirion particles per cell. Luciferase activity was measured 48 h post-infection. Neutralization experiments were performed for each pseudovirion preparation with antibodies against HPV16-L1/L2 (H16.V5) and HPV18-L1/L2 (H18.J4) envelope proteins as indicated. The experiments were done in duplicates, repeated three times and are presented as % of luciferase activity to untreated controls. (**C**) The indicated cell lines were infected with equal amounts of HPV18-PsVs (luc) or HPV16-PsVs (luc), respectively. Luciferase activity was measured 48 h post-infection. The experiments were performed in duplicates, repeated three times and are presented as x-fold increase of luciferase activity to uninfected controls. (**D**) FACS analysis showing ITGA6 expression on the surface of the indicated cell lines. In each panel, the dotted histogram corresponds to the negative control while the solid line shows the histograms of cells stained with the ITGA6 antibody. (**E**) Cells were plated on coverslips and infected with equal amounts of HPV18-PsVs or HPV16-PsVs, respectively, which were labeled with Oregon Green®488 carboxylic acid diacetate succinimidyl ester, a fluorchrome that is only activated after cell entry [43]. The cell line pgsD-677 which is defective in HPV uptake compared to the wildtype CHO-K1 counterpart [50] was included as negative control. Pictures were taken on an Olympus IX81S8F fluorescent microscope at 40x magnification.

In order to elucidate whether inflammation influences HPV infection, we established an in vitro infection assay using HPV pseudovirions that were studied in the context of selected cell lines that were exposed to inflammatory stimuli. We prepared HPV pseudovirions that consisted of the envelope proteins L1 and L2 of HPV18, the HPV type that was mainly found in the HPV positive OSCC biopsies (Figure 1B). Moreover, HPV16-specific PsVs were also prepared so as to test a second oncogenic HPV genotype. Both pseudovirion types encapsidated a luciferase reporter gene plasmid so as to measure PsVs uptake into the cells. SDS-PAGE and neutralisation assays were performed to assess purity and functionality of the pseudovirion preparations. As shown in Figure 2A and B, both HPV18-PsVs (luc) as well as HPV16-PsVs (luc) displayed high purity with only the L1 and L2 protein bands being detectable by SDS-PAGE (Figure 2A). Both the L1 and the L2 protein are about 500 amino acids in length. While the L1 protein has an apparent size of 55 kDa on SDS-PAGE, the size of the L2 protein has been observed to vary slightly between genotypes; the reason for the aberrant mobility of the L2 protein is unknown [47]. When control HaCaT cells, were infected with the HPV18-PsVs, we observed a complete abolishment of infection when pre-incubated with the HPV18-specific neutralising antibody H18.J4 while the HPV16-specific neutralising antibody H16.V5 had no effect (Figure 2B, left panel). Conversely, when HPV16-PsVs were used we observed the opposite effect: the HPV16-specific neutralising antibody H16.V5 inhibited infection while the HPV18-specific neutralising antibody H18.J4 had no effect (Figure 2B, right panel). These assays assured high purity and quality of the respective HPV pseudovirion preparations that were used for all subsequent experiments. To asses HPV pseudovirion infection in selected cell lines, we chose the oesophageal epithelial cell line EPC2-hTERT and the OSCC cell line WHCO1, while HaCaT normal skin keratinocytes and the HPV negative cervical cancer cell line C33A were used as controls of natural HPV infectable cell lines. Infection with HPV18-PsVs (luc) revealed very low infectivity of the two oesophageal cell lines when compared with HaCaT control cells with EPC2-hTERT cells showing almost negligible luciferase activity (Figure 2C, left panel). Similar results were obtained when these cells were infected with HPV16-PsVs (luc) (Figure 2C, right panel). Interestingly, C33A cells showed a much higher luciferase activity when infected with HPV18-PsVs (luc) than with HPV16-PsVs (luc).

The precise mechanism of HPV uptake is still unknown; however, one of the cell surface molecules described as a potential component of the cellular HPV receptor in HaCaT cells is ITGA6 [34,48,49]. We found this receptor to be significantly upregulated in the OSCC tissues compared to corresponding normal tissues (Figure 1A) and investigated whether the differences in HPV pseudovirion infectivity between the cell lines was correlated to the expression of ITGA6. Therefore, we analysed ITGA6 expression in EPC2-hTERT, WHCO1, HaCaT and C33A cells and found that all tested cell lines substantially expressed ITGA6 on their cell surfaces (Figure 2D). These data indicate that the extent of ITGA6 expression could not be correlated to the observed differences in HPV infectivity (Figure 2C).

To further elucidate whether the relatively low luciferase activity detected in the two oesophageal cell lines was due to impaired pseudovirion uptake, PsVs preparations were labelled with Oregon Green®488 carboxylic acid diacetate succinimidyl ester, a fluorochrome that is only activated after cell entry [43]. As shown in Figure 2E, all tested cell lines displayed high intracellular fluorescence with the exception of pgsD-677 cells whose HPV-uptake is known to be greatly reduced compared to their wildtype counterparts CHO-K1 [50]. It appears that EPC2-hTERT and WHCO1 cells do take up the PsVs but do not express the luciferase reporter gene. We confirmed the functionality of the luciferase plasmid by transfection of pGL3 resulting in comparable levels of luciferase activity in the tested cell lines (data not shown).

### *Influence of inflammatory stimuli on HPV pseudovirion uptake* in vitro

**Figure 3:**
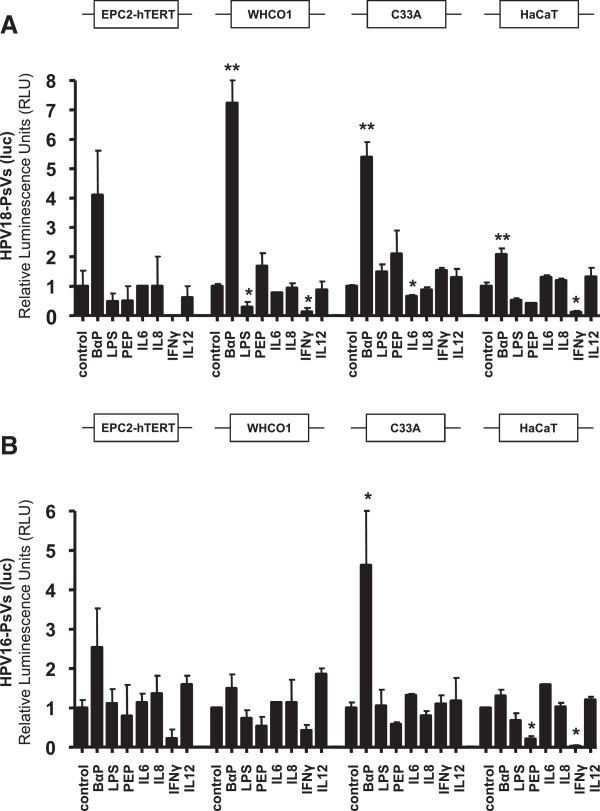
**Influence of inflammatory stimuli on HPV pseudovirion uptake.** The oesophageal epithelial cell line EPC2-hTERT, the OSCC cell line WHCO1 as well as HaCaT and C33A control cells were stimulated with cytokines and irritants for 24 h as indicated, followed by infection with HPV18-PsVs (luc) (**A**) or HPV16-PsVs (luc) (**B**), respectively. Cells were harvested 48 h post-infection and analyzed for luciferase activity. The experiments were done in duplicates, repeated three times and are presented as x-fold increase in luciferase activity to untreated controls. Statistics were done using 2-tailed unpaired *t*-test relative to untreated controls (*, p<0.05; **, p<0.01). Note that the luciferase activity of untreated EPC2-hTERT cells was negligible (Figure 2C); therefore the measured values depicted only reach background activities.

The gene expression data derived from the OSCC biopsies that showed upregulation of IL6 and IL8 and downregulation of IL12A (Figure 1A) were used to mimic inflammation in oesophageal epithelial and cancer as well as control cell lines. Cells were stimulated with IL6, IL8 and IL12 while the anti-viral cytokine IFNy served as negative control. Additionally, the effects of benzo-α-pyrene (BαP), peptidoglycan (PEP) and lipopolysaccharide (LPS) were tested. The appropriate dose and incubation time for every tested drug or cytokine was determined in preliminary experiments for each cell line; shown is the highest concentration of each tested substance that did not affect cell viability during 24 h incubation. Treated cells were infected with HPV-PsVs and analysed for luciferase activity. Except for BαP which significantly and consistently enhanced HPV18-PsVs (luc) reporter gene activity in the tested cell lines, none of the other tested inflammatory stimulants facilitated HPV pseudovirion uptake (Figure 3A). Similar results were obtained for HPV16-PsVs (luc) (Figure 3B), though the effect of BαP was not to the same extent as observed for HPV18-PsVs (luc). Interestingly, the bacteria-derived substances LPS and PEP reduced rather than increased luciferase activity in the various cell lines. This result was not due to cell death as the concentrations used showed no effect of cytotoxicity (not shown). Moreover, while IFNγ drastically reduced HPV pseudovirion infectivity in EPC2-hTERT, WHCO1 and HaCaT cells, C33A cells did not respond to the IFNγ treatment as these cells are known to be defective in IFNγ signalling [51] (Figure 3A and B).

## Discussion

A number of human cancers have an infectious aetiology that have been extensively studied in the past, the best known being the association of HPV with cervical cancer [1]. However, recent studies suggest that viruses may play a more significant role in several other malignancies than the ones with a known viral association. HPV and EBV for example, although widely debated, have been implicated in oesophageal and nasopharyngeal cancers, respectively [10-12,14-16]. While the consequences of viral oncogenes and infection-related inflammation for tumorigenesis have been extensively studied, the impact of tumour-associated inflammation on viral infection is much less understood.

In the present study we chose oesophageal squamous cell carcinoma as an example as this type of cancer is both associated with chronic irritation and inflammation as well as viral infections. Although the role of HPV in the carcinogenesis of OSCC is still debated, a previous study from our laboratory found a high association of HPV11 with OSCC from patients of the Eastern Cape, South Africa [14]. Conducting a similar study with OSCC samples from patients of the Western Cape in South Africa, we here present evidence that underlying infection with HPV plays a minor role as only 9% of the studied patient cohort was infected with HPV with type 18 being the predominant one. Parallel analysis for underlying infection with EBV that served as a technical control for the applied PCR-based detection of viral DNA revealed a slightly higher infection rate of approximately 26%. While the low HPV infection rate was rather unexpected, the number of OSCC patients that were infected with EBV correlates well with the percentage of EBV DNA found in oral squamous cell carcinoma from South African patients ranging from 24% to 27% [52,53]. However, neither virus infection had any significant influence on the expression of the tested inflammatory genes or potential uptake receptors, although statistical analysis was rather limited due to the low number of HPV positive samples. The observed upregulation of IL6 and IL8 and downregulation of IL12A were therefore probably due to tumour-specific immunological responses that were independent of the presence of HPV. The high variability observed between individual samples might be explained by the complexity of the patient cohort and the fact that no personal parameters (such as age, sex, smoking and drinking status, ethnical and social background, stage of tumour etc.) were taken into account in the analysis. However, the large number of patients in the studied cohort allowed us to derive some general conclusion on the significance of the expression profile of the selected genes.

From a technical point of view, detection of HPV sequences in the host genome is rather challenging [54,55]. While various PCR approaches targeting HPV-type specific and HPV-type unspecific mRNA or inserted genomic DNA were tested, including commercially available kits (data not shown), only the widely described nested PCR approach using the primer combination MY09/MY11 followed by GP5+/GP6+ targeting L1, the most conserved protein among papillomaviruses [14,54-57], revealed highest sensitivity and reproducibility. Indeed, only 60-80% of our positive samples could be verified by a given alternative technique while only the combination of all data derived from the various tested HPV detection methods would confirm our (MY09/MY11//GP5+/GP6+)-nested PCR-based results, therefore supporting our data as not being false positives. The primer set MY09/MY11 that uses degenerate primers to detect a wide range of HPV types was not sufficient to pick up any HPV sequences in our patient cohort (data not shown), while a subsequent nested PCR with the GP5+/GP6+ primer set that uses consensus primers with a low annealing temperature to detect a similar range of HPV types resulted in HPV positive tissue samples. However, this approach could still have underestimated HPV prevalence as discussed before [55]. We conclude that both the infection rate as well as the HPV copy number in our patient cohort were very low and probably under the detection level of the particular assay used. The low infection rate indicates that HPV is not a major contributor to the carcinogenesis of OSCC in our patient cohort. This is in agreement with a previous publication that suggests that low HPV copy numbers and infection rates are unlikely to play an essential role in OSCC when compared to cervical cancer [18]. The low number of HPV infected OSCC biopsies was not expected from our previous study [14] but might be explained in part by ethnical parameters and the different living circumstances of the patients in the Eastern versus the Western Cape [58,59]. The Eastern Cape of South Africa is a predominantly poor and underdeveloped province consisting mainly of tribal and informal urban areas with limited access to health services. In contrast to the Western Cape, it has particularly high rates of oesophageal cancer that are attributed to risk factors such as poverty, underdevelopment, poor nutrition and exposure to indoor smoke from combustion of solid fuels [58,60,61]. Although still debated as being the causative agent, it is assumed that HPV infection is only associated with oesophageal cancers originating from high-incidence geographic areas [11,17-20]. This might account for the significantly higher HPV infection rate observed in our previous study [14]; however, infection with the non-oncogenic HPV type 11 might just be interpreted as an indirect measure of poorer environmental conditions.

As HPV infection had no significant influence on inflammation in OSCC we next asked whether the opposite effect played a role, i.e. whether tumour-related inflammation affected the uptake of virus particles. Using HPV as a model, we set up an in vitro infection assay and infected a panel of representative cell lines with HPV-PsVs that were composed of the L1/L2 envelope proteins of HPV18 (as the majority of the HPV positive OSCC biopsies were infected with HPV18) as well as HPV16 (being the most common carcinogenic HPV type worldwide [62,63] and have been reported to be associated with oesophageal cancer [11,17,18]). Surprisingly, we observed no or relatively low HPV pseudovirion infectivity of the immortalised normal oesophageal epithelial cell line EPC2-hTERT and the OSCC cell line WHCO1, respectively, compared to HaCaT or C33A control cells. Although the measured reporter gene activities in EPC2-hTERT cells were negligible, we found that these cells did take up the PsVs. We therefore conclude that these cells might display a non-infectious entry pathway with the virions possibly getting stuck in endocytic vesicles. Unproductive internalization with the hallmark of a stabilised capsid phenotype has been described before [50,64] but was not further investigated in this study as non-infectious uptake routes would not lead to viral oncogene expression, therefore having no relevance for the development of OSCC.

Interestingly, the observed differences in cell line infectivity did not correlate with the presence of ITGA6. Although questioned as a general HPV entry receptor component [24,65], ITGA6 was found to play a role in the uptake of HPV6b L1 pseudovirions into HaCaT cells [34,48] as well as HPV16 L1-VLPs into various cell lines [49]. Moreover, increased expression of ITGA6 was shown to be correlated with the development of oesophageal cancer [66]; and increased aggressiveness, drug resistance and poor prognosis has been correlated with the presence of ITGA6 in leukemia [67]. However, the observed upregulation of ITGA6 in the OSCC tissues as well as its high expression on the surface of the tested cell lines did not correlate with the extent of HPV infectivity. Although we cannot exclude that ITGA6 is involved in HPV uptake, our data indicate that its presence and upregulation in the tumour samples do not play a rate limiting role in the establishment of HPV infection. When the cell lines were stimulated with various inflammatory agents or irritants known to be associated with tumorigenesis of the oesophagus [9], only benzo-α-pyrene facilitated HPV18 pseudovirion (and to a lesser extent HPV16) uptake. BαP, one of the most important carcinogens in tobacco smoke, has long been known to exert a wide range of carcinogenic and pro-inflammatory effects [28,29]. Indeed, both epidemiological evidence as well as in vitro data exist to implicate interactions of HPV and cigarette smoke carcinogens in the progression of cervical cancer [26,27,68]. It was found that BαP increased the infectivity of the oncogenic HPV type 31b but did not directly affect viral gene expression on a transcriptional level [27]. The observed increase in luciferase activity upon BαP treatment might therefore be a result of increased pseudovirion uptake due to altered gene expression of BαP-induced DNA adducts in the host cells [9]. It is unlikely that the BαP-mediated HPV pseudovirion uptake was due to an inflammatory response as none of the tested substances associated with inflammation increased HPV pseudovirion infectivity.

## Conclusion

Derived from the tested parameters, this study indicates that neither tumour-associated inflammation nor viral infection, particularly with HPV, seem to influence each other in OSCC. Further, although highly expressed in the tumour tissues, ITGA6 seems not to be involved in HPV infection due to the low HPV infection rate.

## Abbreviations

HPV: (Human papillomavirus); EBV: (Epstein-Barr virus); OSCC: (Oesophageal squamous cell carcinoma); PsVs: (Pseudovirions); ITGA6: (α6 integrin); luc: (Luciferase); vge: (Viral genome equivalent); BαP: (Benzo-α-pyrene).

## Competing interests

The authors declare that they have no competing interests.

## Authors’ contributions

GS and IMP conceived and designed the study. GS, BvR, SK and MBM performed the experiments. GS analysed the data. GS, LB and IMP contributed reagents and materials. GS wrote the paper. All authors read and approved the final manuscript.

## Pre-publication history

The pre-publication history for this paper can be accessed here:

http://www.biomedcentral.com/1471-2407/13/185/prepub
